# Functional crosstalk between nitrate and ammonium transporters in N acquisition and pH homeostasis

**DOI:** 10.3389/fpls.2025.1634119

**Published:** 2025-06-27

**Authors:** Mikel Rivero-Marcos

**Affiliations:** lnstitute for Multidisciplinary Research in Applied Biology (IMAB), Sciences Department, Public University of Navarre (UPNA), Pamplona, Spain

**Keywords:** ammonium, AMTs, CIPK23, low pH, nitrate signaling, NRT1.1, SLAH3, STOP1

## Abstract

In quantitative terms, nitrogen (N) is the most important essential mineral element for plants, acquired mainly in the form of ammonium (NH_4_^+^) and nitrate (NO_3_^-^). Despite fluctuations in soil NH_4_^+^and NO_3_^-^availability, plants seek to balance their NH_4_^+^-to- NO_3_^-^uptake ratio to avoid the metabolic burden associated with the compensation of an intracellular proton excess or deficit. However, while the molecular mechanisms by which plants mediate and modulate the activity of their uptake systems for NO_3_^-^and NH_4_^+^have been well characterized, it has remained unclear to what extent these transport systems could interact. In this review, the potential contributions of AMTs and NRT1.1 to the overall acquisition of N are highlighted. Both NO_3_^–^independent and -dependent signaling of NRT1.1 in modulating NH_4_^+^tolerance, as well as the underestimated role of AMTs in nutrient and cellular pH homeostasis, are discussed. The interdependency between AMTs and NRT1.1 is considered highly relevant for optimized N uptake in field conditions, where both N forms typically coexist and act complementarily to maintain balanced pH and nutrient homeostasis for optimal plant growth

## Introduction

Among all nutrients, nitrogen (N) is quantitatively the most important essential mineral element for plants and thus its availability a critical determinant in agricultural plant production. The accessibility of N for plant roots varies considerably through space and time, due to soil heterogeneity, anthropogenic N inputs, water flows, microbial activity, and other factors that dislocate or convert N forms (Bloom, 2015). Plants acquire N mostly in the form of ammonium (NH_4_^+^) and nitrate (NO_3_^-^), with the latter being the major form in most plants grown on crop soils (Miller et al., 2007). As plants have adapted to fluctuating NO_3_^-^ concentrations and external pH levels, they evolved sophisticated transport systems that enable efficient NO_3_^-^ uptake coupled with protons (H^+^), ensuring N acquisition even when NO_3_^-^ availability is limited in acidic conditions (Tsai and Schmidt, 2021). At micromolar substrate concentrations, root uptake is mainly mediated by high-affinity NRT2-type transporters (Bouguyon et al., 2012), whereas at millimolar concentrations the high-affinity transporters become repressed and low-affinity transporters take over. Here, the transceptor NRT1.1/NPF6.3/CHL1 (hereby NRT1.1) plays a central role as it confers most of the low-affinity NO_3_^-^ transport capacity (Krouk et al., 2010).

NRT1.1 also mediates NO_3_^-^ signaling and triggers the downstream transcriptional upregulation of a plethora of NO_3_^–^inducible genes (Wang et al., 2009; Bouguyon et al., 2015). Moreover, NRT1.1 is extensively involved in cellular and physiological processes of plant growth and development. These include NO_3_^-^ root-to-shoot translocation, shoot transpiration, auxin transport, seed dormancy relief, and enhanced tolerance to adverse environmental conditions such as H^+^ excess, sodium, cadmium, zinc, lead, or NH_4_^+^ toxicity, as well as iron or phosphorus (Pi) deficiency (Fang et al., 2021). Although specific topics associated with the NO_3_^-^ transport and sensing functions of NRT1.1 have been discussed in multiple reviews, only a few studies have revealed its signaling function in the absence of NO_3_^-^ (Wang et al., 2009; Jian et al., 2018; Hachiya et al., 2011, 2024).

With regard to NH_4_^+^ uptake systems, high-affinity transporters of the AMT family appear being of major importance in plant roots, because NH_4_^+^ concentrations in the majority of crop soils usually range between 20 and 200 μM (Lark et al., 2004). Out of the five AMT proteins expressed in Arabidopsis roots, AMT1.1, AMT1.2, AMT1.3, and AMT1.5, are responsible for high-affinity NH_4_^+^ uptake in N-deficient plants (Loqué et al., 2006; Yuan et al., 2007). Between 60 and 70% of this transport capacity is mediated by AMT1.1 and AMT1.3, while AMT1.2 with its lower affinity confers another approx. 20% and mainly retrieves NH_4_^+^ from the apoplastic transport route (Duan et al., 2018). As concluded from the remaining uptake capacity in the quadruple knock-out line *qko* (*amt1.1*, *1.2*, *1.3*, *2.1*), AMT1.5 confers less than 10% of the overall high-affinity NH_4_^+^ uptake capacity, probably at fairly high affinity (estimated K_m_ ~5 µM (Yuan et al., 2007)). On the other hand, under certain conditions, NH_4_^+^ concentrations can exceed those of NO_3_^-^ and be the main source of N, such as in acidic or paddy soils, which can cause toxicity and arrested plant growth (Xu et al., 2012). Unlike H^+^ cotransport during NO_3_^-^ uptake, only transport activities of AMT1.1 in wheat and common bean are pH-dependent, exhibiting an acid-stimulated regulatory mode (Søegaard, 2009; Ortiz-Ramirez et al., 2011), whereas the transport activities in Arabidopsis, tomato, and rice are pH-independent when expressed in oocytes.

Although the environmental challenge urges to reduce the input of NO_3_^–^based fertilizers (Howarth, 2008), NH_4_^+^ as the main source of N is problematic, and is partly overcome in agricultural practice by the coating of urea fertilizers with urease inhibitors to generate a slow and continuous release of NH_4_^+^. For this reason, the application of both N form in plant species-specific ratios has proven beneficial for crops (Britto and Kronzucker, 2013). However, the reason for NO_3_^-^ and NH_4_^+^ positive synergy in plant growth and development is not fully understood. Furthermore, while the molecular mechanisms by which plants mediate and modulate the activity of their uptake systems for NO_3_^-^ and NH_4_^+^ have been well characterized, it has remained unclear to what extent these transport systems interact to ensure optimal N uptake and growth. In this review, the interdependency of the AMT1s and NRT1.1 during N uptake balance is discussed. Emphasis is placed on how NRT1.1 signaling, both NO_3_^–^dependent and independent, affects NH_4_^+^ tolerance, alongside the underestimated role of AMT-dependent NH_4_^+^ uptake in nutrient homeostasis and cellular pH regulation.

## The regulatory role of NRT1.1 in NH_4_^+^ nutrition

Hachiya and Noguchi (2011), and later Jian et al. (2018), pointed out that a NO_3_^–^independent function of NRT1.1 might exist in Arabidopsis, as functional disruption of NRT1.1 in the so-called *chl1–5* mutant confers increased tolerance to sole NH_4_^+^ nutrition. The main reason for this phenomenon remains unclear. The reduction of shoot transpiration in the *chl1–5* mutant (Guo et al., 2003) was excluded as the cause of the observed NH_4_^+^ tolerance, since no significant differences in the stomatal conductance were found with respect to the wild-type under sole NH_4_^+^ nutrition (Jian et al., 2018).

There is also some divergent findings regarding the possible transcriptional and post-transcriptional repression of AMTs in the *chl1–5* as for its NH_4_^+^ tolerance. On one hand, Hachiya et al. (2011, 2024) did not observe differences in *AMT* transcript levels between wild-type Col-0 and *chl1–5* under sole NH_4_^+^ nutrition. Similarly, Muños et al. (2004) did not find differences in the ^15^NH_4_^+^ uptake capacity under same conditions, leading to the conclusion that NH_4_^+^ tolerance is unlikely to result from *AMT* repression or lower NH_4_^+^ uptake. On the contrary, Jian et al. (2018) observed a significant downregulation of *AMT* genes in the mutant (specifically of *AMT1.1*, *1.3* and *1.5*), together with a decline in the ^15^NH_4_^+^ uptake under sole NH_4_^+^ nutrition. Likewise, they observed a lower accumulation of free NH_4_^+^, which the authors attributed to decreased NH_4_^+^ uptake combined with the induction of glutamine synthetase activity in the roots. They suggested that the enhanced primary assimilation of NH_4_^+^ in *chl1–5* plays a crucial role in its NH_4_^+^ tolerance. The later study by Hachiya et al. (2021) demonstrated that glutamine synthetase activity in roots attenuates NH_4_^+^ toxicity, whereas in shoot causes acidic stress that arrests cell growth in Arabidopsis.

Another process that appears to contribute to mitigating the NH_4_^+^ toxicity in *chl1–5* is the promotion of glucosinolate (GSL) biosynthesis. According to the microarray analysis of Wang et al. (2009), several genes relevant to the aliphatic GSL biosynthetic pathway were induced in *chl1–5* mutant in the absence of NO_3_^-^. Recently, Hachiya et al. (2024) confirmed that the biosynthesis of GSLs and their positive regulator genes *MYB28* and *MYB29* were indeed significantly induced in *chl1–5* in the absence of NO_3_^-^. Since GSLs are N-rich secondary metabolites whose synthesis increases under NH_4_^+^ nutrition (Marino et al., 2016; Coleto et al., 2017), it is plausible that NH_4_^+^ tolerance of *chl1–5* could be due in part to the promotion of NH_4_^+^ assimilation into these compounds rather than into Gln, a H^+^ generating process.

Taken together, despite some discrepancies in *AMT* expression and/or activity, the results suggest that the NH_4_^+^ tolerance observed in *chl1–5* mutants is more closely linked to reduced NH_4_^+^ accumulation in the shoot. However, more studies are needed to elucidate how GSLs, other genes or hormones are involved in the NO_3_^–^independent signaling of NRT1.1 during NH_4_^+^ nutrition.

An additional detail is that the NH_4_^+^-tolerance of *chl1–5* in the absence of NO_3_^-^ was more pronounced at lower pH (Hachiya et al., 2011), so there might be a pH-dependent effect that could ‘amplify’ NRT1.1-dependent signaling. Indeed, excess NH_4_^+^ uptake mediated by AMTs leads to considerable acidification of the apoplastic pH, which causes ionic imbalances during nutrient uptake and an array of constraints to plant fitness in the long-term. Decreasing the medium pH from 6.5 to 5.5 increased the expression and activity of NRT1.1 irrespective of the presence of NO_3_^-^, indicating that pH alone can trigger NRT1.1 readiness in anticipation of reduced levels of available NO_3_^-^ (Tsay et al., 1993; Ye et al., 2021). Although it has not yet been demonstrated which form of NRT1.1 exists predominantly under low pH/high NH_4_^+^ conditions, it is well known that CBL-INTERACTING PROTEIN KINASE 23 (CIPK23) stabilizes the NRT1.1 monomeric state by phosphorylation, enabling NRT1.1 to act as a high-affinity NO_3_^-^ transceptor (Ho et al., 2009; Rashid et al., 2020). Since *CIPK23* is also induced by low pH and excess NH_4_^+^ (see next section) (Straub et al., 2017; Ye et al., 2021), and post-translationally de-repressed by the protein phosphatase ABA-INSENSITIVE 1 and 2 (Ganz et al., 2022; Léran et al., 2015), CIPK23 likely enhances phosphorylation of AMT1 to prevent NH_4_^+^ uptake dependent toxicity, while NRT1.1 to stimulate NO_3_^-^ once available. In addition to CIPK23, the kinase CIPK15 also represses the activity of AMT1.1 in Arabidopsis through phosphorylation under high ammonium conditions (Chen et al., 2020). Also, the phosphatases ABI1 and ABI2 inactivate CIPK15 via dephosphorylation, although there is currently no evidence that they also target NRTs. So far, one could speculate whether might be a ‘competition’ between NRT1.1 and AMTs as common targets for post-translational modifiers (e.g., CIPK23, ABI1, ABI2). This competition could potentially reduce the capacity of those kinases and phosphatases to control their activity, thereby influencing N uptake.

## The other side of the coin: the contribution of AMTs to nutrient and pH homeostasis

In the field, NH_4_^+^ accumulation and low pH often arise simultaneously, because nitrification is generally inhibited under low pH condition. However, strict NH_4_^+^ conditions are unlikely in most crop soils, and even the presence of a low concentration of NO_3_^-^ improves NH_4_^+^ tolerance (Garnica et al., 2010; Hachiya et al., 2012; Zheng et al., 2015; Fang et al., 2016; Du et al., 2021). Thus, under most soil conditions, the growth and tolerance of defected NRT1.1 mutants to NH_4_^+^/low pH are reduced due to their restricted NO_3_^-^/H^+^ uptake and primary NO_3_^-^ response (Fang et al., 2016). At the gene expression level, *NRT1.1* is also induced by low external pH that achieve a maximum at pH 5.0 in Arabidopsis (Ye et al., 2021). A low pH is compensated by more efficient NO_3_^-^ uptake by NRT1.1, whose protonated H356 residue is essential for increasing the NO_3_^-^ and H^+^ cotransport (Sun et al., 2014). Therefore, NRT1.1 is essential to avoid further H^+^ rhizotoxicity, and in turn the associated ionic imbalances such as excessive root accumulation of Fe^2+^ and Mn^2+^, or lower phosphorous (Pi) availability (Kochian et al., 2004).

In the context of NH_4_^+^ nutrition, apoplastic acidification is promoted under excessive NH_4_^+^ nutrition. The AMT-dependent NH_4_^+^ uptake contributes to depolarizing the electrical membrane potential, which in turn enhances net H^+^ efflux, primarily mediated by AHA2 (Meier et al., 2020). Therefore, the induction of *NRT1.1* during NH_4_^+^-promoted apoplastic acidification is plausible for plants to ensure a correct charge balance, which this is in what lies partially the NO_3_^–^dependent alleviation of NH_4_^+^ toxicity when sufficient external NO_3_^-^ is present. Under low external NO_3_^-^ conditions, NRT1.1 is also involved in regulating the apoplastic pH through modulation of AHA2 via the co-receptor kinase QSK1 (Zhu et al., 2024). NRT1.1 interacts with QSK1, prompting QSK1 to phosphorylate AHA2 at the S899 site, which inhibits AHA2 activity and reduces the H^+^ efflux into the apoplast. This mechanism decreases external acidification and is associated with the repression of lateral root (LR) growth under unfavorable low NO_3_^-^ conditions. This functional connection between NRT1.1 and AHA2 extends to the antagonistic role of AMTs, as NH_4_^+^ activates AMT1.3 signaling, promoting LR emergence and higher-order LR branching (Meier et al., 2020). While the role of auxin in NO_3_^–^mediated LR elongation is well established, further research is needed to determine whether AMTs and auxin also contribute to regulating the NRT1.1-QSK1-AHA2 module, particularly under varying N ratios and concentrations.

On top of NRT1.1 function, additional players have been added during adaptation of Arabidopsis to NH_4_^+^ nutrition and low pH, such as the S-type channel SLAH3 (Zheng et al., 2015; Xiao et al., 2022), and the C2H2-type zinc finger transcription factor SENSITIVE TO PROTON RHIZOTOXICITY 1 (STOP1) (Ye et al., 2021). SLAH3 form a functional unit with NRT1.1 and exports chloride and NO_3_^-^ from the cytosol, whereas STOP1 plays a central role in the induction of *NRT1.1* and *CIPK23* as well as in the excretion of organic acids to solubilize external Pi. Hence, SLAH3 and STOP1 link external NH_4_^+^ with the reported efficient of NO_3_^-^ and Pi acquisition, respectively (Tian et al., 2021; Ye et al., 2021). Considering that CIPK23 also phosphorylates NRT1.1, the potassium transporter AKT1, and the metal transporter IRT1, STOP1 appears to be behind in ensuring the nutrient balance and ion homeostasis during excessive NH_4_^+^ nutrition.

Lastly, there are regulatory elements influenced under co-provision of NH_4_^+^, altering the NRT1.1-dependent NO_3_^-^ response, such as NIN LIKE PROTEIN 7 (NLP7) or the POLYADENYLATION FACTOR SUBUNIT 3 (CPSF30). Zhao et al. (2018) revealed that NLP7 acts as a positive regulatory factor upstream of NRT1.1 when NH_4_^+^ is present, modulating the NO_3_^-^ signaling function of NRT1.1. Similarly, the presence of NH_4_^+^ prompts CPSF30 to act upstream of NRT1.1 in NO_3_^-^ signaling without affecting the *CPSF30* expression (Li et al., 2017).

**Figure 1:**
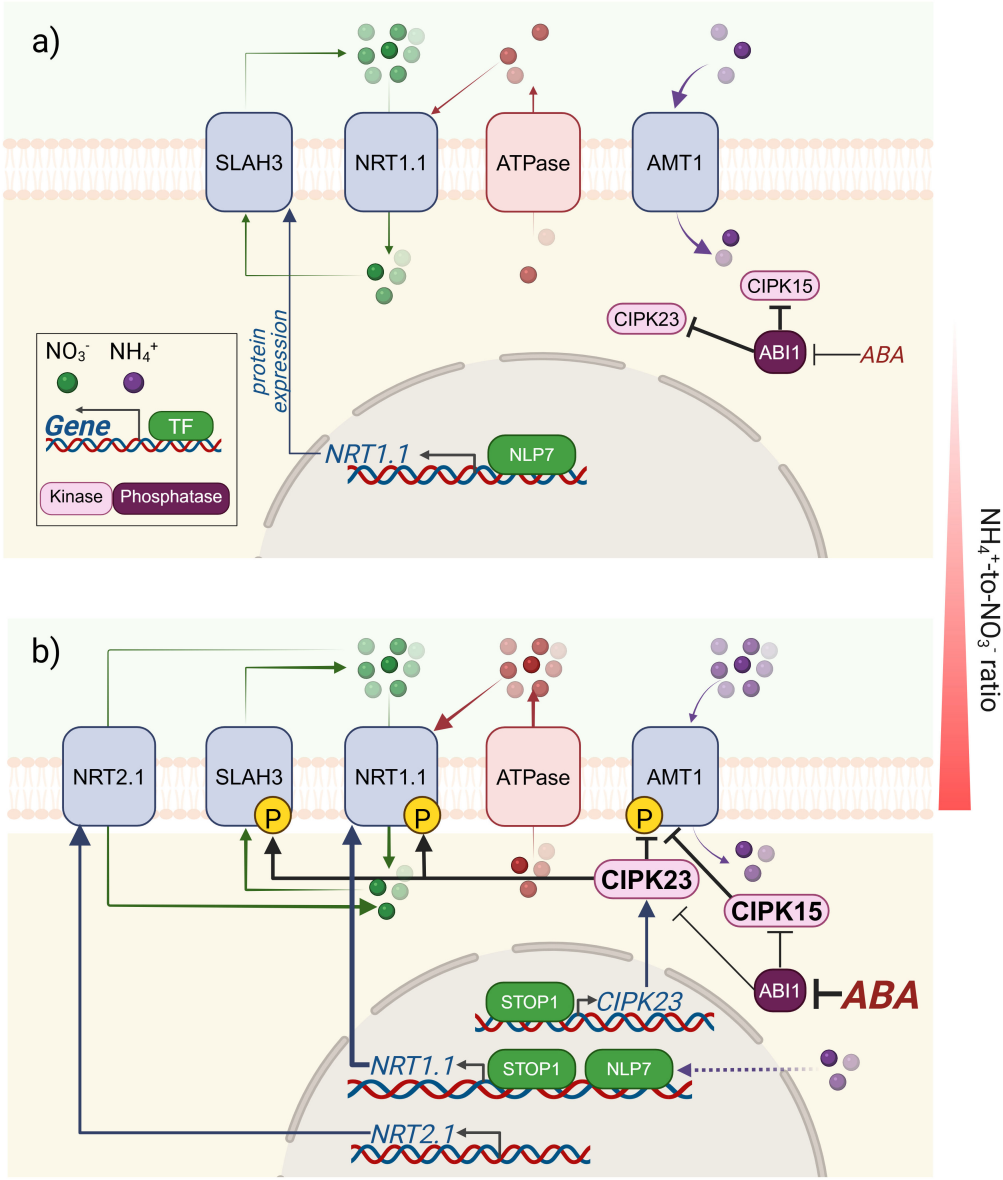
Schematic summary of the common factors that mediate the pH-dependent control of N uptake through NRTs and AMTs. **(a)** Under a balanced NH_4_^+^-to-NO_3_^-^ ratio for proper plant growth, NO_3_^-^ uptake is mainly sustained by NRT1.1, which imposes an as-yet-unclear repression on *NRT2.1*. In addition, in the presence of NH_4_^+^, *NRT1.1* is enhanced by NLP7. In the case of NH_4_^+^, specific uptake occurs through AMTs, and the deactivation of AMTs by phosphorylation is repressed by the phosphatase ABI1. **(b)** As the external NH_4_^+^-NO_3_^-^ ratio increases, it also increases the stress associated with the higher NH_4_^+^-dependent extrusion of H^+^ to the apoplast. Intracellular acidification promotes an enrichment of STOP1 in the nucleus, which induces the transcription of *CIPK23* (the kinase that represses AMT1 activity by phosphorylation), but also induces *SLAH3* and *NRT1.1* to increase NO_3_^-^ efflux/influx as a buffering cycle to alleviate NH_4_^+^-dependent H^+^ stress. ABA accumulation under this unfavorable situation inactivates the phosphatases ABI1 and 2, keeping active the phosphorylating activity of the kinases CIPK15 and CIPK23 towards their common N transporter targets. The figure was created using BioRender.

Although the mechanisms underlying the NO_3_^–^ and NH_4_^+^-dependent sensing function of NRT1.1 remain unclear, it could all respond to a constant fine adjustment in the intracellular pH. In fact, NH_4_^+^ and NO_3_^-^ account for about 70% of the cations and anions taken up by plants, so they can exert a strong influence on intracellular pH despite the strong buffering capacity of the cytosol. The **Figure 1** reveals a complex picture, involving for the first time an interconnection between NRTs and AMTs, whose contribution to regulating pH-dependent mechanisms of N uptake will depend on the balance between nitrate- and ammonium-based nutrition, and vice versa. In the case of NO_3_^-^ nutrition, H^+^ consumption during NO_3_^-^ reduction to nitrite and NH_4_^+^ increases cytosolic pH, and this declines plasma membrane (PM) H^+^-ATPase activity and the consequent respiratory expense. Therefore, cotransport of H^+^ together with a cytosolic pH increase during NO_3_^-^ uptake and reduction contributes to overall plant cell alkalinization. On the other hand, the decrease in pH driven in part by the AMTs-mediated NH_4_^+^ uptake enrich the transcription of key players that activates not only the expression of *NRT1.1*, but also earlier genes involved in the acquisition of other nutrients whose availability or acquisition is particularly sensitive to external pH changes. For instance, Pi uptake is also an anion/H^+^ cotransport process and the second most important macronutrient for plants, aside from its close relationship with Fe availability and homeostasis. While, under low Pi conditions, the repression of *NRT2*s by NIGTs might be part of an adaptive response to reduce energy consumption and maintain the ionic balance, AMTs are upregulated to promote the NH_4_^+^ uptake and subsequent H^+^ release. For this reason, the AMT1 family is considered to play a role in local Pi signaling, and potentially increases Pi solubility in the medium (Paz-Ares et al., 2022). The discovery of NRT1.1 and AMT-dependent regulation of N and P-related genes may lead to a deeper understanding of the strategies employed by plants to adapt to changing nutrient environments.

## New perspectives

To date, attempts to enhance N use efficiency in crops through modulation of *AMT* gene expression have achieved limited success, likely due to NH_4_^+^ sensitivity. This phenomenon represents a bottleneck in improving NH_4_^+^-based nutrition, as NH_4_^+^ when supplied as the predominant N form proves highly deleterious for most crops (Britto and Kronzucker, 2002). Notably, NH_4_^+^ sensitivity exhibits substantial interspecies and intraspecies variability among cultivars, reflecting an intrinsic ecophysiological adaptation (Rivero-Marcos et al., 2024), where the role of AMTs in NH_4_^+^ acquisition and signaling/adaptation may also vary substantially across plant systems.

In contrast, NO_3_^-^ transporters have been more thoroughly characterized, owing partly to their broader functional diversity across plant species (Ruffel et al., 2025). This disparity is not surprising given the predominance of NO_3_^-^ as the primary inorganic N form in aerobic soils - a consequence of microbial competition for reduced N compounds, agricultural tillage practices, and other edaphic and climate factors (Mao et al., 2025). The nitrate transporter NRT1.1 has evolved dual functionality as a NO_3_^-^ sensor, like the TF NLP7, and the overexpression of these components enhances growth and N use efficiency in several species (e.g., *AtNRT1.1*, Sakuraba et al., 2021; *AtNLP7*, Yu et al., 2016; *OsNRT1.1A*, Hu et al, 2015; *OsNRT1.1B*, Wang et al., 2018).

A key unresolved question concerns the synergistic growth response observed when NO_3_^-^ and NH_4_^+^ are co-supplied. Remarkably, even micromolar NO_3_^-^ concentrations (≤100 μM) - considered non-nutritional – to NH_4_^+^-fed plants can significantly alleviate NH_4_^+^ toxicity and stimulate growth in some NH_4_^+^-preferring species (Garnica et al., 2009; Yan et al., 2023). This strongly implicates the primary NO_3_^-^ response (PNR) pathway in growth optimization, and likely includes downstream AMT-related components. Consequently, classical PNR components like NRT1.1, but also potentially AMTs - which may serve dual roles as NH_4_^+^ transporters and sensors (Pflüger et al., 2024) - represent promising targets for crop improvement strategies. Future research should investigate kinases and phosphatases regulated by NO_3_^-^ signaling that may modulate AMT activity, and potential physical interactions between NRT1.1 and AMTs, which is plausible given the known ‘promiscuity’ among N transporters and key proteins regulating N uptake (Zhu et al., 2024).

Here, it has been shown how AMTs and NRTs sustain the vital uptake of N, thus affecting the N use efficiency of the plant and cellular pH homeostasis. While AMT-dependent NH_4_^+^ uptake largely contributes to apoplast and rhizosphere acidification, NO_3_^-^ and other nutrient uptake such as Pi becomes more efficient through pH-dependent induction of TFs like *STOP1*. NRT1.1 has the most essential contribution not only in NO_3_^-^ acquisition and signaling, but also in modulating NH_4_^+^ response in the absence of NO_3_^-^. Therefore, understanding the mechanisms by which plants regulate N acquisition in dependence or not of NRT1.1, and evaluating the contribution of AMTs to changes in internal and external pH, is critical for improving N use efficiency and nutrient uptake across diverse species and crop systems. These insights indicate that conventional approaches examining the responses to NO_3_^-^ and NH_4_^+^ separately, or after N deprivation, are no longer sufficient. Field-relevant advances will require integrated approaches using both N forms and corresponding mutants, capitalizing on their synergistic potential to achieve growth optimization and yield improvement under reduced N inputs.
